# Assessing Therapeutic Response to Radium-223 with an Automated Bone Scan Index among Metastatic Castration-Resistant Prostate Cancer Patients: Data from Patients in the J-RAP-BSI Trial

**DOI:** 10.3390/cancers15102784

**Published:** 2023-05-16

**Authors:** Kazuhiro Kitajima, Junpei Kuyama, Takashi Kawahara, Tsuyoshi Suga, Tomoaki Otani, Shigeyasu Sugawara, Yumiko Kono, Yukihisa Tamaki, Ayumi Seko-Nitta, Yoshinobu Ishiwata, Kimiteru Ito, Akira Toriihara, Shiro Watanabe, Makoto Hosono, Hideaki Miyake, Shingo Yamamoto, Ryohei Sasaki, Mitsuhiro Narita, Koichiro Yamakado

**Affiliations:** 1 Department of Radiology, Hyogo Medical University, Hyogo 663-8131, Japan; ko-yamakado@hyo-med.ac.jp; 2 Nuclear Medicine, Chiba Cancer Center, Chiba 260-8717, Japan; jkuyama@chiba-cc.jp; 3 Department of Urology and Renal Transplantation, Yokohama City University Medical Center, Kanagawa 232-0024, Japan; takashi_tk2001@yahoo.co.jp; 4 Department of Radiology, Kobe City Medical Center General Hospital, Hyogo 650-0047, Japan; tsuyoshi_suga@kcho.jp; 5 Diagnostic Imaging and Nuclear Medicine, Kyoto University Graduate School of Medicine, Kyoto 606-8303, Japan; totani@kuhp.kyoto-u.ac.jp; 6 Advanced Clinical Research Center, Fukushima Medical University, Fukushima 960-1295, Japan; shige-s@fmu.ac.jp; 7 Department of Radiology, Kansai Medical University, Osaka 573-1191, Japan; kohnoy@hirakata.kmu.ac.jp; 8 Department of Radiation Oncology, Faculty of Medicine, Shimane University, Shimane 693-0021, Japan; ytamaki@med.shimane-u.ac.jp; 9 Department of Radiology, Shiga University of Medical Science, Shiga 520-2192, Japan; ayumis@belle.shiga-med.ac.jp; 10 Department of Radiology, Yokohama City University Hospital, Kanagawa 236-0004, Japan; ishi_y@yokohama-cu.ac.jp; 11 Department of Diagnostic Radiology, National Cancer Center Hospital, Tokyo 104-0045, Japan; kimito@ncc.go.jp; 12 PET Imaging Center, Asahi General Hospital, Toyama, 939-0741, Japan; toriiharaa@hospital.asahi.chiba.jp; 13 Department of Nuclear Medicine, Hokkaido University Hospital, Hokkaido 060-8648, Japan; shirow@med.hokudai.ac.jp; 14 Department of Radiation Oncology, Faculty of Medicine, Kindai University, Osaka 577-8502, Japan; hosono@med.kindai.ac.jp; 15 Department of Urology, Hamamatsu University School of Medicine, Shizuoka 431-3125, Japan; hmiyake@hama-med.ac.jp; 16 Department of Urology, Hyogo Medical University, Hyogo 663-8131, Japan; shingoy@hyo-med.ac.jp; 17 Department of Radiation Oncology, Graduate School of Medicine, Kobe University, Hyogo 650-0017, Japan; rsasaki@med.kobe-u.ac.jp; 18 Department of Urology, Shiga University of Medical Science, Shiga 520-2192, Japan; mnari@belle.shiga-med.ac.jp

**Keywords:** bone scintigraphy, castration-resistant prostate cancer (CRPC), overall survival, ^223^Radium-dichloride, treatment response

## Abstract

**Simple Summary:**

This study was a retrospective investigation of a Japanese cohort of 205 metastatic castration-resistant prostate cancer (mCRPC) patients who received Ra-223 in 14 hospitals between July 2016 and August 2020 and for whom bone scintigraphy before and after the radium-223 treatment was available. Following treatment, alkaline phosphatase (ALP) decline (%ALP < 0%) was noted in 72.2% (148/205), automated bone scan index (aBSI) decline (%aBSI < 0%) in 52.7% (108/205), and PSA decline (%PSA < 0%) in 27.8% (57/205). Furthermore, a reduction in both aBSI and ALP was seen in 87 (42.4%), a reduction in only ALP was seen in 61 (29.8%), a reduction in only aBSI was seen in 21 (10.2%), and in both aBSI and ALP increasing/stable (≥0%) was seen in 36 (17.6%) patients. Multiparametric analysis showed changes in PSA (HR 4.30, 95% CI 2.32–8.77, *p* < 0.0001), aBSI (HR 2.22, 95%CI 1.43–3.59, *p* = 0.0003), and ALP (HR 2.06, 95%CI 1.35–3.14, *p* = 0.0008) as significant prognostic factors for OS. For mCRPC patients treated with Ra-223, aBSI change is useful as an imaging biomarker for treatment response assessment and survival prediction.

**Abstract:**

To evaluate the usefulness of change in the automated bone scan index (aBSI) value derived from bone scintigraphy findings as an imaging biomarker for the assessment of treatment response and survival prediction in metastatic castration-resistant prostate cancer (mCRPC) patients treated with Ra-223. This study was a retrospective investigation of a Japanese cohort of 205 mCRPC patients who received Ra-223 in 14 hospitals between July 2016 and August 2020 and for whom bone scintigraphy before and after radium-223 treatment was available. Correlations of aBSI change, with changes in the serum markers alkaline phosphatase (ALP) and prostate-specific antigen (PSA) were evaluated. Additionally, the association of those changes with overall survival (OS) was assessed using the Cox proportional-hazards model and Kaplan–Meier curve results. Of the 205 patients enrolled, 165 (80.5%) completed six cycles of Ra-223. Following treatment, ALP decline (%ALP < 0%) was noted in 72.2% (148/205), aBSI decline (%aBSI < 0%) in 52.7% (108/205), and PSA decline (%PSA < 0%) in 27.8% (57/205). Furthermore, a reduction in both aBSI and ALP was seen in 87 (42.4%), a reduction in only ALP was seen in 61 (29.8%), a reduction in only aBSI was seen in 21 (10.2%), and in both aBSI and ALP increasing/stable (≥0%) was seen in 36 (17.6%) patients. Multiparametric analysis showed changes in PSA [hazard ratio (HR) 4.30, 95% confidence interval (CI) 2.32–8.77, *p* < 0.0001], aBSI (HR 2.22, 95%CI 1.43–3.59, *p* = 0.0003), and ALP (HR 2.06, 95%CI 1.35–3.14, *p* = 0.0008) as significant prognostic factors for OS. For mCRPC patients treated with Ra-223, aBSI change is useful as an imaging biomarker for treatment response assessment and survival prediction.

## 1. Introduction

Radium-223 dichloride (Ra-223), a targeted alpha emitter, selectively binds to high-bone turnover sites caused by bone metastasis [1]. Alpha particles with a very short range (<100 μm) primarily induce double-stranded DNA breaks, resulting in highly localized cytotoxic effects with lower levels of damage to surrounding tissues. The ALSYMPCA trial showed both the efficacy and safety of Ra-223 in metastatic castration-resistant prostate cancer (mCRPC) patients with symptomatic bone metastasis [2]. The results of the randomized phase 3 ALSYMPCA study, which compared patients with CRPC and symptomatic bone metastasis who received the best standard of care plus Ra-223 with those who received a placebo, showed that the former group had a median overall survival (OS) prolonged by 3.6 months [hazard ratio (HR) 0.70. 95% confidence interval (CI) 0.58–0.83, *p* < 0.001, median 14.9 vs. 11.3 months]. Additionally, the results indicated that Ra-223 treatment was well tolerated and resulted in a low incidence of grade 3 or 4 myelosuppression (Ra-223 vs. placebo: anemia 13% and 13%, neutropenia 2% and 1%, thrombocytopenia 7% and 2%).

For a proper evaluation of response to Ra-223 treatment, as well as stratification of responders and non-responders for subsequent therapy, the determination of radiographic response biomarkers is necessary. While alkaline phosphatase (ALP) and prostate specific antigen (PSA) levels are typically monitored [3], response biomarkers that provide better accuracy for choosing the most appropriate course of treatment would be helpful. Bone scintigraphy findings of mCRPC patients with bone metastasis, in whom bone uptake is proportional to bone remodeling activity, are fundamental for monitoring treatment in mCRPC cases and recommended by the Prostate Cancer Clinical Trials Working Group (PCWG3) for estimating skeletal response to therapy [4]. Nevertheless, the response to Ra-223 shown by bone scintigraphy and the correlation with clinical outcome factors have yet to be thoroughly elucidated.

To quantify the extent of skeletal tumor burden shown by bone scintigraphy as a percentage of total skeletal weight, an automated bone scan index (aBSI) (%) has been developed [5]. The important information it provides makes it a potentially helpful tool for estimating the total quantitative skeletal metastatic burden in mCRPC patients. This automated methodology utilizes artificial intelligence and has a rapid processing time, while it has also been demonstrated to provide accurate and reproducible results [6]. In a prospective phase III study of 721 mCRPC patients treated at multiple institutions recently reported, aBSI was clinically validated as a prognostic biomarker [7]. Additionally, several prior evaluations of pretreatment aBSI as a prognostic imaging biomarker for mCRPC patients who received Ra-223 treatment showed a significant association of baseline values with OS [8,9,10,11,12,13,14]. On the other hand, data regarding response biomarkers to evaluate treatment response or non-response shown by aBSI in mCRPC patients treated with Ra-223 are scarce. Two studies demonstrated that change in aBSI was a useful finding for survival prediction [10,12], whereas two others found no significance related to aBSI change [9,11]; thus, its usefulness remains controversial.

The Japanese Ra-223 Therapy in Prostate Cancer using Bone Scan Index (J-RAP-BSI) multicenter trial was performed to examine the effectiveness of aBSI as an imaging biomarker for the evaluation of bone metastasis in CRPC patients in Japan receiving Ra-223 therapy [14]. The present multicenter retrospective study was conducted as a sub-analysis of results obtained in that trial to evaluate aBSI findings for assessment of response to such therapy as well as survival prediction in mCRPC patients.

## 2. Materials and Methods

### 2.1. Ethics

The institutional review board at the Hyogo College of Medicine (number 3662, on 17 December 2020) and 13 hospitals (Chiba Cancer Center, Yokohama City University Medical Center, Kobe City Medical Center General Hospital, Kyoto University Graduate School of Medicine, Fukushima Medical University Hospital, Kansai Medical University, Shimane University Faculty of Medicine, Shiga University of Medical Science, Yokohama City University Hospital, National Cancer Center Hospital, Asahi General Hospital, Hokkaido University Graduate School of Medicine, Kindai University) approved this retrospective J-RAP-BSI trial collecting clinical data of patients receiving Ra-223 therapy and bone scintigraphy. The requirement for written informed consent for study participation was waived by the institutional ethics committee. Instead, participants were given an opportunity to opt-out if they did not want their information to be used in this study. This study was conducted in accordance with the principles of the Declaration of Helsinki.

### 2.2. Study Design and Patient Selection

We conducted a total of 258 patients with mCRPC and bone metastasis treated at 14 different medical institutions, including Hyogo College of Medicine Hospital, Chiba Cancer Center, Yokohama City University Medical Center, Kobe City Medical Center General Hospital, Kyoto University Graduate School of Medicine, Fukushima Medical University Hospital, Kansai Medical University, Shimane University Faculty of Medicine, Shiga University of Medical Science, Yokohama City University Hospital, National Cancer Center Hospital, Asahi General Hospital, Hokkaido University Graduate School of Medicine, and Kindai University Hospital. Each underwent bone scintigraphy between July 2016 and August 2020 as part of the J-RAP-BSI trial before starting Ra-223 treatment. Patients who underwent technetium-99m methylene diphosphonate bone scintigraphy (^99m^Tc-MDP; PDRadiopharma, Inc., Tokyo, Japan) examinations within two months before the first Ra-223 cycle and again within two months after the final Ra-223 cycle with results available for analysis were included in this study. Skeletal disease burden was classified according to the Soloway classification [15], while aBSI was determined using the specifically developed BONENAVI^®^ software package, version 2 (PDRadiopharma, Inc., Tokyo, Japan) [16]. Ra-223 therapy consisting of a total of six intravenous injections (55 kBq/kg body weight) administered every 28 days was given to mCRPC patients without disease progression, findings showing unacceptable toxicity, declining performance status, or by the request of the patient. The present cohort consisted of males >18 years old, with bone metastasis, no visceral metastasis or lymph node ≥3 cm, hemoglobin >8.4 gm/dl, white blood cell count >1.5 × 10^9^, and platelet count >100 × 10^9^ at the time of the initial Ra-233 injection. Androgen deprivation therapy was continued during Ra-233 therapy, while other medications, such as denosumab or bisphosphonates, were prescribed at the discretion of the attending physician. Previous systemic therapy, such as abiraterone, enzalutamide, docetaxel, or cabazitaxel, was permitted for inclusion, while concomitant treatment with abiraterone or chemotherapy was not. Patients were excluded if they had undergone chemotherapy within the previous four weeks, showed impaired kidney or liver function, or were affected by inflammatory bowel disease. Finally, 205 patients who met the study criteria were considered eligible for the evaluations.

### 2.3. Evaluations

Hematologic parameters (ALP, PSA) were evaluated before each Ra-223 administration. Bone scintigraphy to evaluate aBSI was performed before treatment and at its discontinuation, with the percentage change in those values calculated. The association of percentage changes in ALP and PSA with that of aBSI was evaluated to determine prognostic value. The Common Terminology Criteria for Adverse Events (CTCAE) package, version 4.03, was used for grading AEs [17]. The assessment of toxicity was performed at each treatment cycle.

### 2.4. Statistical Analysis

Spearman’s rank correlation coefficient was used to assess the relationships of percentage changes in ALP and PSA with aBSI. Correlation strength was categorized using conventional statistical criteria, with 0–0.19 regarded as very weak, 0.2–0.39 as weak, 0.40–0.59 as moderate, 0.6–0.79 as strong, and 0.8–1 as very strong.

For evaluating the prognostic value of changes in ALP and PSA, and also aBSI, univariate and multivariate analyses were performed using a Cox proportional hazard model with stepwise regression. Any change in those parameters from the baseline was categorized as a decrease or increase/no change. To estimate survival distribution, the Kaplan–Meier product limit was used. A log-rank test was utilized for survival difference analysis.

Data analyses were performed using SPSS (version 21.0) and all tests were two-sided. *p* values < 0.05 were considered to indicate statistical significance.

## 3. Results

### 3.1. Patient Characteristics

Detailed baseline characteristics of the patients are presented in Table 1. At the initial Ra-223 treatment, the median age was 73 years (54–88 years). Prior to starting Ra-223 therapy, 58 patients had undergone external radiotherapy for bone metastasis, 158 received novel androgen receptor-targeted agent therapy with enzalutamide and/or abiraterone, 91 received taxane-based chemotherapy with docetaxel and/or cabazitaxel, and 144 received bisphosphonate/denosumab treatment. Additionally, 57 patients received enzalutamide and 120 bisphosphonate/denosumab during Ra-223 therapy.

AEs were found to occur in 120 (58.5%) patients, with major AEs, such as anemia (28.3%), thrombocytopenia (13.2%), impaired liver function (8.8%), neutropenia (7.8%), and general fatigue (6.3%), noted regardless of grade. G3-G4 toxicity developed in 12 patients (5.9%), including anemia, general fatigue, and bone pain in three patients each, pancytopenia in two, and hyponatremia in one.

Six cycles of Ra-223 were given to 165 patients (80.5%), while 12 (5.9%) received five cycles, nine (4.4%) received four, 13 (6.3%) received three, three (1.5%) received two, and three (1.5%) received one. The reasons for early discontinuation were progression (*n* = 23), hematologic toxicity (*n* = 5), declining performance status (*n* = 6), bone pain (*n* = 5), and patient request (*n* = 1). 

### 3.2. Changes in Serum Markers ALP and PSA, and aBSI

The median ALP level before starting Ra-223 therapy was 285 U/L [range 68–3494 U/L, mean ± standard deviation (SD) 396.0 ± 375.5 U/L] and after finishing therapy was 223 U/L (range 67–4333 U/L, mean ± SD 321.8 ± 380.4 U/L). Additionally, the median change in ALP was −16.3% (range −91.4% to 314.9%, mean ± SD −9.71 ± 51.1%). A decrease in ALP was observed in 148 (72.2%) of the 205 patients. 

Median PSA before Ra-223 therapy was 15.48 ng/mL (range 0.002–3630.56 ng/mL, mean ± SD 79.7 ± 286.9 ng/mL) and after therapy was 26.65 ng/mL (range 0.002–4908.02 ng/mL, mean ± SD 217.2 ± 575.1 ng/mL), while the median change in PSA was 102.1% (range −100% to 999.9%, mean ± SD 226.5 ± 334.3%). A decreased PSA level was observed in 57 (27.8%) patients.

**Figure 1:**
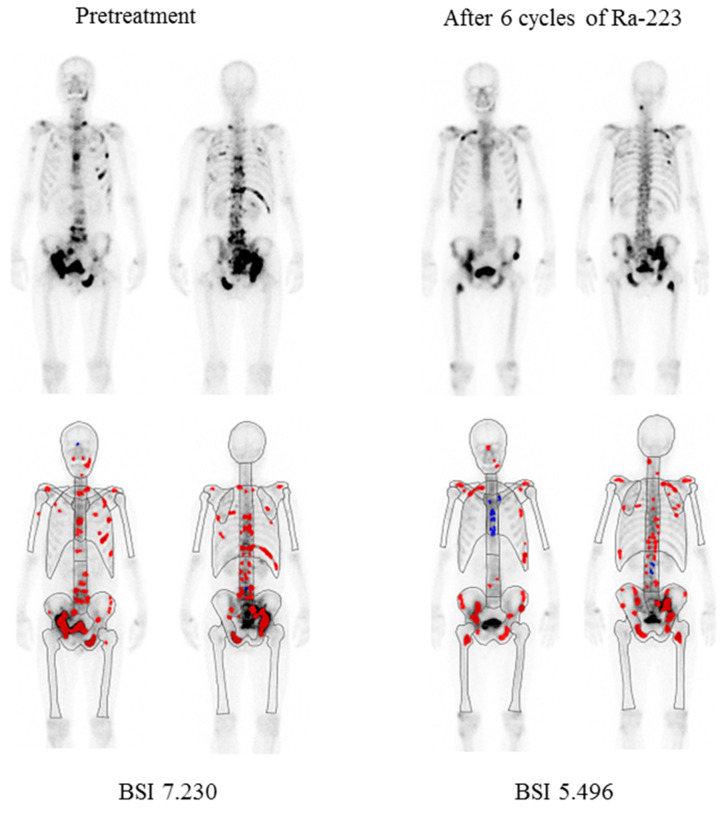
Representative bone scintigraphy findings of a 68-year-old male. Illustration of change in automated bone scan index (aBSI) after six cycles of Ra-223. Red color represents hotspots detected using the BONENAVI software package and included in aBSI assessment.

The median aBSI level prior to beginning Ra-223 therapy was 1.07 (range 0.02–18.58, mean ± SD 2.42 ± 3.28), while after therapy was 1.05 (range 0.0–13.38, mean ± SD 2.60 ± 3.17). The median change was −4.55% (range −100% to 995.8%, mean ± SD 56.1 ± 195.6%) and a decreased aBSI level was noted in 108 (52.7%) of the 205 patients. Details of a representative case are presented in Figure 1.

### 3.3. Correlation of Changes in Serum Markers ALP and PSA with aBSI

**Figure 2:**
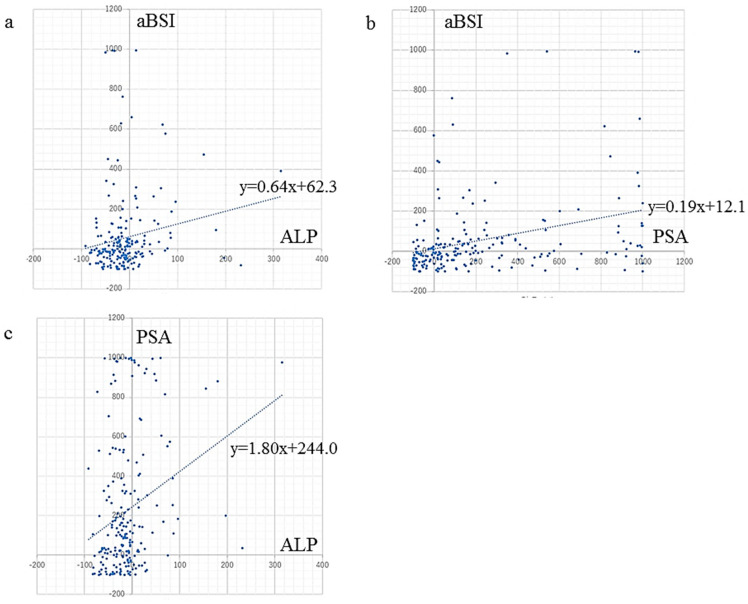
Correlation between changes in serum markers ALP and PSA, and rate of aBSI change. aBSI change showed (**a**) a very weak but significant correlation with ALP change (r = 0.17, *p* = 0.017), and (**b**) a weak but significant correlation with PSA change (r = 0.33, *p* < 0.0001). (**c**) ALP change showed a weak but significant correlation with PSA change (r = 0.27, *p* < 0.0001).

aBSI change (%) showed a very weak but significant correlation with ALP change (r = 0.17, *p* = 0.017) and a weak but significant correlation with PSA change (r = 0.33, *p* < 0.0001) (Figure 2). 

Eighty-seven patients (42.4%) showed reductions in both aBSI and ALP (<0%), while 61 (29.8%) showed only reduced ALP (%ALP < 0%) and 21 (10.2%) only reduced aBSI (%aBSI < 0%). In contrast, 36 (17.6%) patients showed increases/stable in aBSI and ALP (≥0%). A concordant response regarding aBSI and ALP change was observed in 123 patients (60.0%).

There were 43 patients (21.0%) with reductions in both aBSI and PSA (<0%), 14 (6.8%) with only reduced PSA (%PSA < 0%), and 65 (31.7%) with only reduced aBSI (%aBSI < 0%), while an increase/stable in both aBSI and PSA (≥0%) was seen in 83 (40.5%). One hundred twenty-six patients (61.5%) showed a concordant response regarding aBSI and PSA change. 

Additionally, a weak but significant correlation of ALP change with PSA change was noted (r = 0.27, *p* < 0.0001) (Figure 2). Forty-six patients (22.4%) showed reductions in both ALP and PSA (<0%), while 102 (49.8%) showed only reduced ALP (%ALP < 0%) and 11 (5.4%) only reduced PSA (%PSA < 0%). In contrast, 46 (22.4%) patients showed increases/stable in both ALP and PSA (≥0%). A concordant response regarding ALP and PSA changes was observed in 92 patients (44.9%).

Among three parameters (aBSI, ALP, and PSA), 36 patients (17.6%) showed reductions (<0%) in all aBSI, ALP, and PSA, while 69 (33.7%) showed two reduced parameters (<0%), and 69 (33.7%) only one parameter. In contrast, 31 (15.1%) patients showed increases or stable (≥0%) in all three parameters. A concordant response regarding the change of three parameters was observed in 67 patients (32.7%).

### 3.4. Survival Analysis

After a median period of 21.1 months (range 2.8–62.9 months, mean ± SD 24.2 ± 14.6 months) from the initial Ra-223 administration, 90 (43.9%) of the 205 patients died from prostate cancer. Univariate analysis showed that all analyzed parameters, including change in ALP (*p* = 0.028), PSA (*p* < 0.0001), and aBSI (*p* < 0.0001), were significant prognostic factors for OS (Table 2). Multivariate analysis indicated that ALP (HR 2.06, 95%CI 1.35–3.14, *p* = 0.0008), PSA (HR 4.30, 95%CI 2.32–8.77, *p* < 0.0001), and aBSI (HR 2.22, 95%CI 1.43–3.59, *p* = 0.0003) changes were significant prognostic factors related to OS (Table 2).

**Figure 3:**
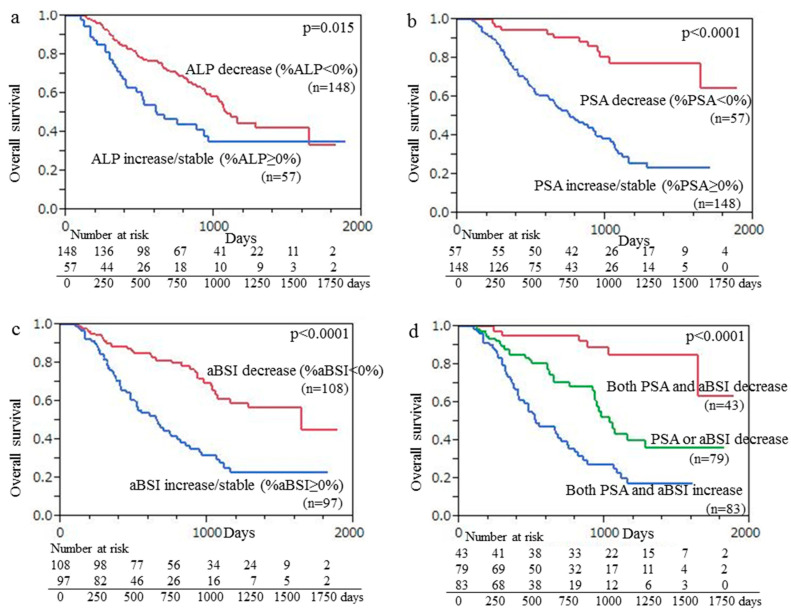
Kaplan–Meier curve findings showing overall survival (OS) following initiation of Ra-223 therapy based on change of (**a**) ALP, (**b**) PSA, and (**c**) aBSI. (**a**) Median OS of 148 patients with ALP decline was significantly longer than that of 57 patients with ALP increase/no change (22.6 vs. 15.8 months, *p* = 0.015). (**b**) Median OS of 57 patients with PSA decline was significantly longer than that of 148 patients with PSA increase/no change (32.5 vs. 16.8 months, *p* < 0.001). (**c**) Median OS of 108 patients with aBSI decline was significantly longer than that of 97 patients with aBSI increase/no change (27.1 vs. 16.2 months, *p* < 0.001). (**d**) Median OS of the 43 patients with declines in both aBSI and PSA (33.3 months) was significantly longer (*p* < 0.001) than that of the 79 patients with a decline in only aBSI or PSA (20.5 months) and that of the 83 patients with aBSI increase/no change and PSA increase/no change (15.2 months).

A Kaplan–Meier curve was used, which demonstrated that the median OS for the 148 patients with ALP decline (median OS months) was significantly greater than that of the 57 patients with an increase or no change in ALP (22.6 vs. 15.8 months, *p* = 0.015), as shown in Figure 3a. Kaplan–Meier curve findings also demonstrated that the median OS of the 57 patients with PSA decline was significantly longer than that of the 148 patients with an increase or no change in PSA (32.5 vs. 16.8 months, *p* < 0.001), with those results shown in Figure 3b. Additionally, the median OS of the 108 patients with aBSI decline was significantly longer than that of the 97 patients with an aBSI increase or no change (27.1 vs. 16.2 months, *p* < 0.001) (Figure 3c). Finally, the median OS of the 43 patients with declines in both aBSI and PSA (33.3 months) was significantly longer (*p* < 0.001) than that of the 79 patients with a decline in only aBSI or PSA (20.5 months) and that of the 83 patients with aBSI increase/no change and PSA increase/no change (15.2 months) (Figure 3d).

## 4. Discussion

Although there are inherent limitations of a retrospective review of patient data, the present multi-center cohort of mCRPC cases that underwent Ra-223 therapy, and also pre- and post-treatment bone scintigraphy examinations is the largest such group reported, with the findings considered useful for analysis of the real-world impact of Ra-223. It was clarified that ALP change is very useful as a marker to evaluate Ra-223 treatment response, while PSA change is a very useful marker for prognosis prediction, and aBSI change is very useful for both evaluating treatment response and predicting the prognosis of treated patients. Furthermore, to the best of our knowledge, this study is the first to evaluate correlations of changes in three biomarkers (ALP, PSA, and aBSI). The results showed relatively low correlations among those, though they might be useful to indicate Ra-223 treatment response.

Decreases in ALP, PSA, and aBSI were observed in 72% (148/205), 28% (57/205), and 53% (108/205), respectively, of the present Ra-223-treated cases, similar to data presented in the Anand study, which showed those reductions in 81% (54/67), 13% (9/67), and 36% (24/67), respectively [12]. Similarly, Prelaj et al. [18] reported that decreases in ALP and PSA following Ra-223 therapy were observed in 78% (25/32) and 59% (19/32), respectively, of their cases. In the ALSYMPCA trial, 47% showed an ALP response and 16% a PSA response (≥30% reduction from baseline) [2]. It has also been demonstrated that ALP, a biomarker of osteoblast activity, is superior to PSA as a biomarker of Ra-223 efficacy [10,18,19,20]; thus, that has emerged as the leading biomarker in treated patients. An increase in PSA can be the result of the development of lymph node or visceral metastasis, which is not affected by Ra-223. Based on the results obtained in the present study and the natural target of Ra-223, the systemic bone scan parameter aBSI should be considered as the main parameter in association with ALP when evaluating response to radionuclide treatment.

Whether ALP change is an accurate biomarker for predicting survival remains controversial. One study demonstrated that an ALP response ≥30% or ≥10% reduction from the baseline value in patients receiving Ra-223 therapy can be a good predictor of OS [21], whereas three others found that ALP response after Ra-223 was not effective for OS prediction [10,12,19]. In another study, Sartor et al. [22] showed that ALP decline at 12 weeks after the first Ra-223 administration was correlated with OS, though the results did not meet the statistical requirements to indicate significance. However, the PSA level does not provide accurate information to elucidate the extent of skeletal metastasis or the effects of treatment on bone disease progression, though the recommended regimen does provide a survival benefit [23]. Moreover, Prelaj et al. [18] found that PSA response was significantly associated with survival, similar to the present findings, while two other groups [10,12] also reported univariate and multivariate analysis results showing that aBSI change was significantly useful for predicting survival.

When monitoring the effectiveness of therapy using assessments of clinical, biochemical (e.g., PSA, ALP), and imaging findings in prostate cancer patients treated with androgen deprivation therapy, cytotoxic chemotherapy, hormone therapy, and palliative radiotherapy, as well as Ra-223 therapy [24,25,26,27,28,29], care must be taken regarding the so-called “flare effect”, which results from an early or transient rise in PSA level or tracer uptake during a bone scan procedure, followed by a later decline, especially in the early phase of treatment. Bone flare phenomena related to bone scintigraphy during the first three months of Ra-223 treatment have been reported [24,25,26]. Keizman et al. [26] also noted that a transient increase in bone metastasis-related pain was observed in 27% of their analyzed patients, while bone scintigraphy findings showed an increase in the number of bone lesions at three months when compared with the baseline in 26% of those cases and in 6% at six months as compared with three months. Castello et al. [27] reported PSA flare in 9.5–35.7% of prostate cancer patients treated with Ra-223, while better OS was noted in mCRPC patients experiencing PSA flare during Ra-233 therapy as compared to those with a progressive PSA increase. Following treatment, other imaging tools, such as fluorodeoxyglucose-positron emission tomography (FDG-PET), prostate-specific membrane antigen (PMSA)-PET, and fluoride-PET, can be useful to assess tumor load reduction, particularly in patients showing increased PSA, which is helpful for differential diagnosis between progression and pseudo-progression (related to PSA flare phenomenon) [27,28,29]. Based on their findings, Castello et al. [27] strongly recommended that Ra-223 therapy should not be discontinued after an early and transient PSA rise. In particular, physicians should be aware of the possibility of PSA flare induced by 233RaCl2 therapy during at least the first two months of treatment and that it does not represent a sign of disease progression.

A single-center study found that aBSI calculation derived using the DASciS software package, developed by the Sapienza University of Rome, is useful for predicting survival in mCRPC candidates treated with Ra-223 [11], which was also shown by the results of a multicenter study conducted in Italy [30]. This package has been demonstrated to be a simple tool that requires no more than a single demonstrative training session for a participating center. After gaining experience, the estimated time for a single BSI calculation is less than one minute, which is significantly lower than the time required for manual BSI calculation.

This study has some limitations, including its retrospective nature and inclusion of results obtained at multiple institutions. Therefore, generalization of the findings is limited and statistical errors are possible. Additionally, following the failure of Ra-223 therapy, the modalities used for the next chosen treatment were not uniform and various methods were used according to the choice of the attending physician. To more accurately clarify the usefulness of aBSI as an imaging biomarker for the evaluation of the response to Ra-223 therapy in clinical settings, a prospective multicenter trial that includes a larger cohort will be necessary.

## 5. Conclusions

The present findings indicate that the change in aBSI shown by bone scintigraphy is accurate and reliable for use as an imaging biomarker to assess treatment response in mCRPC patients treated with Ra-223, as well as to predict prognosis. Its use may provide for better management of mCRPC patients undergoing Ra-223 therapy.

## Figures and Tables

**Table 1:** Baseline patients’ characteristics.

Characteristics	Value (Range)	%
Median age (years)	73 (54–88)	
Initial stage		
II	19	9.3
III	29	14.1
IV	157	76.6
Initial median PSA (ng/mL)	94.9 (0.103–22412)	
Initial Gleason score		
6	6	29.3
7	23	11.2
8	50	24.4
9	97	47.3
10	29	14.1
Prior external radiotherapy for primary prostate cancer	50	24.4
Prior external radiotherapy for bone metastasis	58	28.3
Prior ARTA therapy	158	77.1
Prior taxane-based chemotherapy	91	44.4
Prior systemic therapy line for mCRPC		
0	8	3.9
1	26	12.7
2	125	61.0
3	28	13.7
4	18	8.8
Prior bisphosphonate/denosumab	144	70.2
ECOG performance status		
0	131	63.9
1	65	31.1
2	7	3.4
3	2	0.96
Median Hemoglobin (g/dl)	12.5 (8.4–18.1)	
Median Neutrophil count (/μL)	4127 (1512–12470)	
Median Platelet count (×10^4^/μL)	22.3 (10.1–53.4)	
Median ALP (U/L)	285 (68–3494)	
Median LDH (U/L)	201 (100–952)	
Median PSA (ng/mL)	15.48 (0.002–3630.56)	
Extent of skeletal disease		
<6 metastases	77	37.6
6–20 metastases	63	30.7
>20 metastases	57	27.8
Superscan	8	3.9
Median aBSI	1.07 (0.02–18.58)	
Number of ^223^Ra applications		
1 injection	3	1.5
2 injections	3	1.5
3 injections	13	6.3
4 injections	9	4.4
5 injections	12	5.9
6 injections	165	80.5
Concomitany use of enzalutamide	57	27.8
Concomitany use of Bisphosphonate/denosumab	120	58.5

PSA: prostate specific antigen, ARTA: novel androgen receptor-targeted agents, mCRPC: metastatic castration-resistant prostate cancer, ECOG: Eastern Cooperative Oncology Group, ALP: alkaline phosphatase, LDH: lactate dehydrogenase, PSA-DT: PSA doubling time, aBSI: automated bone scan index.

**Table 2:** Univariate and multivariate Cox proportional hazards regression model of predicting OS.

Item	Number of	Univariate Analysis		Multivariate Analysis	
	Patients	*p* (Log-Rank)	Hazard Ratio (95% CI)	*p* (Log-Rank)	Hazard Ratio (95% CI)
ALP					
Decrease (%ALP < 0%)	148	0.028	1.72 (1.09–2.64)	0.0008	2.06 (1.35–3.14)
Increase/stable (%ALP ≥ 0%)	57				
PSA					
Decrease (%PSA < 0%)	57	<0.0001	5.20 (2.86–10.4)	<0.0001	4.30 (2.32–8.77)
Increase/stable (%PSA ≥ 0%)	148				
aBSI					
Decrease (%aBSI < 0%)	108	<0.0001	2.94 (1.92–4.60)	0.0003	2.22 (1.43–3.50)
Increase/stable (%aBSI ≥ 0%)	97				

CI: confidence interval, ALP: alkaline phosphatase, PSA: prostate specific antigen, aBSI: automated bone scan index.

## Data Availability

The data presented in this study are available in this article.

## Abbreviations

aBSI	automated bone scan index
mCRPC	metastatic castration-resistant prostate cancer
OS	overall survival
HR	hazard ratio
CI	confidence interval
ALP	alkaline phosphatase
PSA	prostate-specific antigen
PCWG3	Prostate Cancer Clinical Trials Working Group3
^99m^Tc-MDP	technetium-99m methylene diphosphonate
CTCAE	Common Terminology Criteria for Adverse Events
SD	standard deviation
FDG	fluorodeoxyglucose
PET	positron emission tomography
PSMA	prostate-specific membrane antigen
